# Mapping Nutritional Assessment and Management in Oncology: Challenges and Perspectives from a Regional Survey

**DOI:** 10.3390/nu18152433

**Published:** 2026-07-25

**Authors:** Elisa Mattavelli, Emanuele Cereda, Alessandro Amorosi, Saba Ancillai, Anna Boggio, Marco Danova, Gabriella Farina, Monica Giordano, Patrizia Gnagnarella, Elisa Merelli, Giulia Mulazzani, Paolo Pedrazzoli, Piero Rivizzigno, Alessandro Scardoni, Alessandro Zucchelli, Riccardo Caccialanza, on behalf of the Clinical Nutrition Network of Lombardy

**Affiliations:** 1 Clinical Nutrition and Dietetics Unit, Department of Oncology, Comprehensive Cancer Center, Fondazione IRCCS Policlinico San Matteo, 27100 Pavia, Italy; e.mattavelli@smatteo.pv.it (E.M.);; 2 Department of Internal Medicine and Medical Therapy, University of Pavia, 27100 Pavia, Italy; p.pedrazzoli@smatteo.pv.it; 3 Welfare General Directorate, Regione Lombardia, 20124 Milan, Italy; 4 School of Medicine, Vita-Salute San Raffaele University, 20132 Milan, Italy; 5 Clinical Nutrition and Dietetics Unit, ASST Fatebenefratelli Sacco, 20154 Milan, Italy; 6 Department of Internal Medicine and Medical Oncology, ASST of Pavia, 27100 Pavia, Italy; 7 Medical Oncology Unit, ASST Fatebenefratelli Sacco, 20154 Milan, Italy; 8 Medical Oncology Unit, ASST Lariana, 22074 Como, Italy; 9 Division of Epidemiology and Biostatistics, IEO European Institute of Oncology IRCCS, 20141 Milan, Italy; 10 Clinical Nutrition and Dietetics Unit, ASST Melegnano-Martesana, 20077 Melegnano, Italy; 11 Clinical Nutrition and Dietetics Unit, ASST Santi Paolo e Carlo, 20146 Milan, Italy; 12 Department of Oncology, Comprehensive Cancer Center, Fondazione IRCCS Policlinico San Matteo, 27100 Pavia, Italy; 13 Codice Viola, Pancreatic Cancer Advocacy Group, 23880 Lesmo, Italy; 14 Clinical Nutrition Unit, ASST Grande Ospedale Metropolitano Niguarda, 20162 Milan, Italy; 15 Department of Oncology and Hematology-Oncology, University of Milan, 20122 Milan, Italy

**Keywords:** malnutrition, oncology, nutritional screening, nutritional assessment, therapeutic care pathway, regional survey

## Abstract

Background: Malnutrition is common among patients with cancer and is associated with poorer clinical outcomes and increased healthcare costs. However, nutritional care remains suboptimal in routine oncology practice. This study aimed to assess the implementation of nutritional screening, assessment and management across oncology centers in Lombardy (Italy) and to identify critical areas to support the development and implementation of a regional Diagnostic–Therapeutic Care Pathway (PDTA). Methods: A 21-item questionnaire developed by the Oncology Commission of the Clinical Nutrition Network of Lombardy Region was administered to all regional oncology centers. Data were collected via the Welfare General Directorate platform from 13 November 2024 to 31 December 2024, and analyzed in aggregate form. Results: In total, 55 out of 59 centers responded (93.2%). Nutritional care was available in 78% of centers. Systematic nutritional risk screening was reported by 42% of centers, while 51% performed a comprehensive nutritional assessment in all patients with a positive screening result. Nutritional assessment was scheduled at both initial and follow-up visits in 53% of centers. In the perioperative setting, prehabilitation programs were available in 49% of centers, and preoperative immunonutrition was administered in 35%. Tools for assessing patient-reported outcomes and quality of life were available in only 15% and 20% of centers, respectively. Conclusions: The survey suggests some improvements compared to previous Italian surveys but highlights persistent gaps in the organization and delivery of nutritional care for patients with cancer.

## 1. Introduction

Malnutrition is common among cancer patients, with prevalence estimates ranging from 20% to 70% [1]. In oncology, malnutrition has been associated with reduced treatment tolerance [2], a higher risk of complications [3,4,5], poorer prognosis and survival [3,4,6,7], lower quality of life [8], prolonged hospital stays [9], and increased healthcare costs [10]. Malnutrition may occur at any stage of the disease and, if unrecognized and untreated, may progressively worsen. Therefore, current guidelines recommend early and repeated nutritional screening and assessment throughout the cancer care pathway with regular follow-up and reassessment to monitor changes in nutritional status and guide timely nutritional interventions [11,12,13].

Despite increasing awareness of the detrimental effects of untreated malnutrition, its management remains heterogeneous across European and Italian oncology settings [14,15,16]. A retrospective analysis of administrative datasets from three European countries (France, Germany, and Italy) showed that cancer-related malnutrition is frequently unrecognized and untreated, while clinical nutrition is often initiated at the end of life or not prescribed at all [14]. Similarly, two nationwide Italian surveys conducted in 2016 and 2020 revealed that, although oncologists and patients recognized the relevance of malnutrition and nutritional support in the cancer care continuum, nutritional care practices were largely inadequate [15,16]. These surveys also highlighted the need for shared regional and national care pathways to strengthen nutritional care in oncology.

In response, Lombardy Region introduced several initiatives to improve the prevention and management of malnutrition. Regional Decree No. 14890 of 18 October 2022, of the Lombardy Region established the Clinical Nutrition Network, with the aim of promoting a homogeneous regional approach to malnutrition management [17]. Subsequently, Resolution No. 1812 of 29 January 2024, of Lombardy Region mandated systematic nutritional screening for all patients admitted to Oncology, Geriatrics, Internal Medicine, General Surgery, Gastroenterology, Nephrology, Cardiology, and Pediatrics wards. Moreover, nutritional screening was included among the mandatory requirements for hospital facilities and Integrated Home Care services [18].

Although nutritional risk screening and assessment are essential components of nutritional care in oncology, they should be embedded within structured pathways that ensure timely access to comprehensive assessment and appropriate nutritional support when needed [19]. Based on this need, the Lombardy Region has been developing a Diagnostic–Therapeutic Assistance Pathway (PDTA = Percorso Diagnostico Terapeutico Assistenziale) to define a homogeneous, structured, and simplified pathway for nutritional care in oncology, together with the standards required for its implementation across regional centers [20].

This survey aimed to describe the current state of nutritional care in oncology centers of Lombardy Region to identify key areas for improvement in support of PDTA implementation. These included timely and systematic nutritional risk screening, access to equipment for nutritional assessment, adequate staff training, and support for patients and caregivers, and perioperative nutritional care.

## 2. Materials and Methods

The Oncology Commission of the Clinical Nutrition Network of Lombardy developed a 21-question multiple-choice questionnaire (Table S1). Before dissemination, the questionnaire was reviewed by an independent panel of experts, including oncologists and specialists in clinical nutrition. The items were discussed and refined to ensure adequate coverage of the main domains of nutritional care in oncology.

The questionnaire addressed the availability and organization of nutritional care services and their integration into oncology practice (Questions [Q] 1 and 2); nutritional care in the perioperative setting (Q3 and Q4); the timing and modalities of nutritional risk screening and referral pathways (Q5–Q8) and nutritional assessment and follow-up procedures (Q9–Q13). The final section explored the use of patient-reported outcomes (PROs), quality of life (QoL) assessment (Q14 and Q18), and perceived satisfaction (Q19), as well as educational initiatives for patients, caregivers, and healthcare professionals (Q15, Q16, Q17, and Q21). Internal quality-control measures, including audits and the evaluation of the effectiveness and safety of nutritional care, were also assessed (Q20).

To facilitate clarity and consistency of responses across centers, the questionnaire included multiple-choice response options and was administered through a single institutional web-based platform. All oncology centers in the Lombardy Region (*n* = 59) were invited to participate. Formal invitations were sent by email to the managers of the individual centers. The survey was available through a dedicated webpage on the institutional portal of Lombardy Region, active from 13 November 2024–31 December 2024.

The survey collected only institutional and organizational information at the healthcare facility level. No patient-level data or sensitive personal data were collected. The questionnaire was completed non-anonymously at the institutional level, as responses needed to be linked to the participating healthcare facilities to monitor participation and assess completeness of data. Data were subsequently analyzed and reported in aggregate form.

Quality checks were conducted to identify any inconsistencies in survey responses. No centers were excluded, but some missing responses were present. Missing data were retained as not available and are reported in Table S1 where applicable. For the main survey questions, data are presented as both numbers and percentages relative to the total number of participating centers. For sub-items, percentages were calculated using the denominator relevant to the corresponding main question. Centers were categorized according to their institutional status (public vs. private). Comparative analyses between groups were performed using chi-square or Fisher’s exact test for categorical variables. Comparisons between public and private centers were exploratory and intended to identify potential differences in the organization and delivery of nutritional care across healthcare settings. No formal adjustment for multiple comparisons was applied. Statistical analyses were performed using IBM SPSS Statistics, version 30.0 (IBM Corp., Armonk, NY, USA).

## 3. Results

Detailed responses to the full survey questionnaire are provided in Table S1.

**Figure 1:**
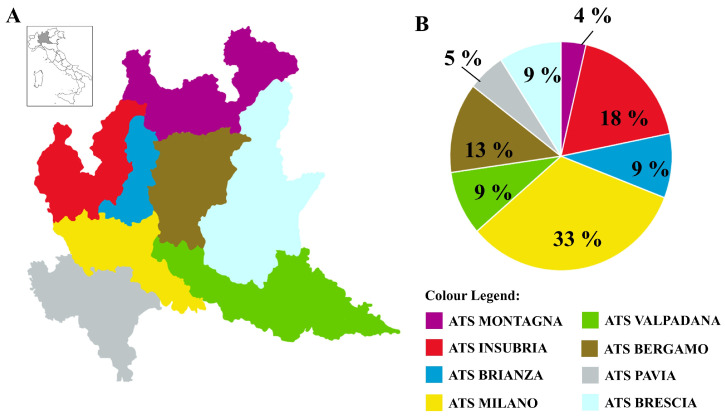
Territorial distribution of the oncology centers responding to the survey. (**A**) Geographical distribution of the participating centers across the eight Local Health Agencies (Agenzie di Tutela della Salute, ATS) of the Lombardy Region. (**B**) Proportional distribution of participating centers by ATS, expressed as percentages.

Of the 59 oncology centers in Lombardy Region, 55 responded to the survey (93.2%). Responding centers were located across all Local Health Agencies (ATS = Agenzie di Tutela della Salute) in the Lombardy Region (Figure 1A,B). Of the responding centers, 62% were public healthcare facilities within the Italian National Health System, whereas 38% were private facilities.

### 3.1. General Information (Questions 1 and 2)

Overall, 78% of centers (*n* = 43) reported providing nutritional care to patients with cancer. A total of 31 oncology centers (56%) reported having a procedure describing the nutritional care pathway for patients with cancer. Among these centers, 24 (77%) reported that the procedure was fully implemented and 22 (71%) reported that it included criteria for referral to the multidisciplinary team evaluation. A positive nutritional risk screening result was the most frequently reported criterion (10/22, 45%).

### 3.2. Surgery Setting (Questions 3 and 4)

Regarding perioperative care, nutritional prehabilitation programs were available in 27 oncology centers (49%) and preoperative immunonutrition was administered in 19 out of 55 oncology centers (35%). Exploratory comparisons according to institutional status showed that public centers more frequently implemented nutritional prehabilitation programs compared to private facilities (62% vs. 29%, *p* = 0.026), whereas the use of preoperative immunonutrition was reported in 53% of public centers and 5% of private facilities (*p* < 0.001) (Table S2).

### 3.3. Screening and Access Methods for Nutritional Assessment (Questions 5 to 8)

Systematic nutritional risk screening for all patients with cancer was reported by 23 oncology centers (42%). Repeated screening during hospitalization or outpatient visits was reported by 17 centers (31%). Exploratory comparisons according to institutional status showed that repeated nutritional risk screening was reported more frequently by public centers than by private facilities (respectively 41% vs. 14%, *p* = 0.042) (Table S2). Overall, for patients with a positive nutritional risk screening, referral to the clinical and dietetic unit or to the nutritional team was most commonly managed by telephone and/or email (40% of cases). A computerized referral system was used by 12 centers (22%) while 7 (12%) centers reported using both computerized and telephone/email-based modalities. Exploratory comparisons according to institutional status showed that public centers more frequently used computerized referral modalities whereas private facilities more often relied on telephone calls or emails (*p* < 0.001) (Table S2).

For patients at severe nutritional risk, the reported time for referral for nutritional care was within 24 h in 9 centers (16%), within 48 h in 23 centers (42%), within 72 h in 11 centers (20%) and more than 72 h in 12 centers (22%). Exploratory comparisons according to institutional status showed shorter reported times to referral for nutritional care in public centers than private facilities (*p* < 0.001) (Table S2).

### 3.4. Nutritional Assessment (Questions 9 to 13)

A total of 28 oncology centers (51%) reported performing a comprehensive nutritional assessment for all patients with a positive nutritional risk screening. In the remaining 27 centers (49%), nutritional assessment was performed according to other criteria, most commonly clinical evaluation (12 centers, 44%) or the presence of weight loss and/or dysphagia (7 centers, 26%).

Nutritional assessment was scheduled at both the first and follow-up visits in 29 oncology centers (53%). In 10 centers (18%), nutritional assessment was scheduled at the first visit and repeated only if weight loss occurred. In the remaining centers, assessments were performed either at the first visit (5 centers, 9%) or only when patients reported weight loss (11 centers, 20%). In 35 centers (64%), the first nutritional visit was guaranteed within one month from cancer diagnosis.

Exploratory comparisons according to institutional status showed that public centers more frequently reported structured scheduling including both initial and follow-up visits, whereas private centers more often performed assessments only in selected cases (*p* < 0.001) (Table S2). Telemedicine or teleconsultation for follow-up visits was available in 11 oncology centers (20%). Equipment required for nutritional risk screening and assessment was available in 44 centers (80%).

### 3.5. Patient-Reported Outcomes (PROs), Quality of Life (QoL), and Patient and Staff Training (Questions 14 to 21)

Tools for collecting nutrition-related patient-reported outcomes (PROs) were unavailable in 47 oncology centers (85%), whereas validated quality-of-life assessment tools were unavailable in 44 centers (80%). Patient satisfaction was assessed through specific tools in 39 centers (71%).

About half of the oncology centers (53%, *n* = 29) reported providing informational materials with preliminary nutritional recommendations to patients at the time of diagnosis, and 64% (*n* = 35) implemented nutritional education interventions for patients and caregivers since early phases of the treatment process. In addition, 38 oncology centers promoted initiatives aimed at raising awareness of healthy eating habits and lifestyles. Oncology staff training activities were reported to be performed in 40 oncology centers (73%). Most oncology centers (*n* = 47, 85%) did not report conducting periodic internal audits of nutritional care.

No significant differences were observed between public and private centers in the availability of equipment, telemedicine use, staff training, patient education, or quality-related indicators (Table S2).

## 4. Discussion

Although nutritional care was available in a substantial proportion of responding centers, our survey revealed considerable heterogeneity and several gaps in its organization and delivery in northern Italy (Lombardy Region). Despite the growing awareness of the clinical relevance of cancer-related malnutrition, our findings are consistent with previous evidence showing that nutritional risk screening, nutritional assessment, and the provision of appropriate nutritional support remain suboptimal in oncology [15,16].

The response rate in the present survey (93.2%) was higher than the previous Italian surveys. Indeed, the survey performed in 2016 among AIOM members had a 5.7% response rate [15], and the survey performed in 2020, involving Italian oncology centers and FAVO members, had a response rate of 51.6% and 38.5%, respectively [16]. The high response rate may reflect a growing interest in the nutritional management of patients with cancer among oncology centers in the Lombardy Region. Over the past few years, the Lombardy Region has implemented several initiatives to enhance the nutritional management of patients with cancer. Following the establishment of the Clinical Nutrition Network in 2022 [17], systematic nutritional risk screening was introduced as mandatory [18]. Although these measures represent an important starting point, they should be implemented uniformly and coherently across all regional healthcare settings. For this reason, a regional PDTA was developed [20] to define structured and shared pathways for managing cancer-related malnutrition. This survey aimed to provide key elements for the PDTA implementation. The first step in nutritional care is the early identification of patients at nutritional risk, enabling the timely initiation of tailored nutritional support [19]. This approach may prevent further nutritional deterioration and improve nutritional status [21], treatment tolerance [22,23], survival [24,25], and quality of life [21]. Beyond the clinical advantages, the implementation of systematic nutritional screening and timely support may lead to substantial economic benefits, helping to reduce the additional costs associated with malnutrition, thus contributing to a more efficient use of healthcare resources [10]. Current European and Italian guidelines recommend that nutritional risk screening should be performed systematically in all cancer patients at the time of diagnosis, within 48 h since hospital admission [11,12,13,19]. Nutritional risk should be reassessed at each access during the treatment phase and follow-up, and whenever symptoms develop and/or nutritional status worsens [11,12,13,19]. Screening should be based on simple, quick, easy-to-use and validated tools that can also be administered by healthcare professionals without specific training in clinical nutrition [26,27,28]. Instruments such as the Nutritional Risk Screening 2002 (NRS-2002) [27], and the Malnutrition Universal Screening Tool (MUST) [28] meet these requirements and are recommended by current clinical guidelines for the early identification of patients at nutritional risk [11,12,13,19]. However, our data showed that systematic nutritional risk screening was reported by only 23 oncology centers (42%). In addition, only 31% of the centers reported repeating nutritional screening during hospitalization or outpatient follow-up visits. When stratified by institutional status, public centers reported more frequent implementation of repeated nutritional risk screening than private facilities. This finding may suggest differences in the integration of screening procedures into routine clinical practice across healthcare settings. Since this survey was administered before the mandatory nutritional screening resolution [18], these findings are likely to reflect a pre-policy scenario. Future studies will be needed to assess whether this measure will promote the systematic implementation of nutritional risk screening and reduce the variability observed across public and private centers. Beyond the implementation of screening itself, effective nutritional care management requires that patients identified as being at risk are promptly referred for comprehensive nutritional assessment, including body composition evaluation when appropriate, and for tailored nutritional support delivered by a clinical nutrition service or appropriately trained healthcare professionals [11,12,13].

Our data indicate that only 28 oncology centers (51%) performed a comprehensive nutritional assessment in all patients with a positive nutritional screening result. In the remaining centers, a nutritional assessment was performed according to other clinical criteria, most commonly clinical evaluation or in the presence of weight loss and/or dysphagia. Overall, these findings indicate that both systematic screening and the subsequent assessment of patients identified as being at nutritional risk remain incompletely integrated into routine oncology care across Lombardy oncology centers. A similar pattern has been described in previous Italian surveys [15,16].

More recently, the survey conducted by the NutriOnc Research Group reported a 71% rate of nutritional screening administration. However, the systematic nature of the screening provision was not investigated. Additionally, they reported that in 91.8% of cases, a positive screening result led to patients’ referral for nutritional assessment. Nevertheless, these findings should be interpreted with caution due to the low response rate (73 responders out of 350 invited), which may limit the generalizability of the results [29].

Compared with previous Italian surveys, we detected improvements in the programming of nutritional assessments. In fact, it emerged that 53% of oncology centers planned nutritional assessment both at first and follow-up visits. This percentage is higher compared to those previously reported (21% and 14–27% in 2016 and 2020, respectively) [15,16]. Nevertheless, the percentage regarding the scheduling of nutritional assessment visits suggests substantial opportunities for improvement, especially to ensure that patients receive scheduled follow-ups for nutritional assessment. In this case, the use of computerized systems can play a crucial role in managing clinical information, promoting adherence to therapies, providing decision-making support, and monitoring the care of cancer patients [30]. However, only 20% of respondents have taken advantage of telemedicine and teleconsultation systems. In this context, the co-occurrence of more frequent computerized referral modalities and shorter reported times to referral for nutritional care in public centers may indicate a potential advantage of more structured digital referral pathways. However, this observation should be interpreted cautiously and does not establish a causal relationship.

In the present survey, we also focused on the surgical setting. The evidence supports the beneficial effects of preoperative immunonutrition on clinical outcomes. Particularly, malnourished patients receiving immunonutrition experience shorter hospital stays and reduced post-operative complications [31,32,33,34]. Our data indicate that 49% of centers reported offering a prehabilitation program before surgery, and 35% reported administering preoperative immunonutrition. The moderate adoption rates observed in our data reflect ongoing practices [35]. When stratified by institutional status, public centers more frequently implemented prehabilitation programs and preoperative immunonutrition compared to private facilities. Clinicians may selectively implement these programs, considering factors such as patient nutritional status, cancer type, and surgical procedure. European and Italian guidelines include all oncological patients undergoing curative or palliative surgery in the Enhanced Recovery After Surgery (ERAS) program, which encompasses screening for malnutrition and support for those considered at nutritional risk [13,36]. Oral/enteral immunonutrition is advised for patients with upper gastrointestinal tract cancer undergoing surgical treatment [13,36].

Within this framework, it is important to underline that a robust multidisciplinary approach, in which dietitians, physicians, nurses, pharmacists, and other specialists actively collaborate to address malnutrition, remains a cornerstone for effective nutritional risk management [37].

Following the Shared Care Plan for Home Artificial Nutrition, approved in the Lombardy Region by Welfare Decree No. 14274, dated 25 October 2021, multidisciplinary nutritional teams (MNTs) were established in hospitals that lacked structured Clinical Nutrition Units (CNUs) [38]. In our survey, 71% of centers with a standardized procedure referred patients with cancer at nutritional risk to a multidisciplinary discussion.

The involvement of multiple healthcare professionals in the management of patients with nutritional needs highlights the importance of specialized training or systems that facilitate information exchange and coordination of care.

In recent years, QoL and PROs have gained increasing attention as key components of patient-centered cancer care [39], highlighting the need to implement tools for their routine assessment [40]. However, these tools were available in only a minority of Lombardy Region oncology centers, with only 15% reporting the use of PROs and 20% the use of validated QoL scales. The use of these tools may provide valuable data to evaluate the individual patient’s nutritional care pathways, further personalize nutritional support based on individual needs, and improve the quality of interaction between clinicians and patients [41].

Patient engagement also begins with adequate information. The last section of the survey investigated the presence of information and educational initiatives for patients and their caregivers. Approximately 60% of oncology centers provided nutritional education already in the initial phases of therapy, and 53% provided nutritional informative materials. Active patient involvement through appropriate education may enhance adherence to the therapeutic program and facilitate timely recognition of specific nutritional needs, thereby making patients active and autonomous in the nutritional care process [42,43].

From a broader perspective, further implementation of structured nutritional care in oncology may be supported by reinforcing systematic nutritional screening through its integration into clinical workflows, strengthening multidisciplinary collaboration and staff training, and expanding the use of digital tools for follow-up and monitoring. In addition, the routine adoption of patient-reported outcomes and quality of life measures, together with regular audit processes, may help improve the quality and consistency of care.

While our survey, conducted through a dedicated webpage, achieved broad reach across the region’s cancer centers (93.2%), some limitations should be acknowledged. First, the web-based format may have exposed the survey to a potential digital divide, although the high response rate suggests that this issue had a limited impact on overall participation. Moreover, the relatively brief data collection period (13 November 2024–31 December 2024), may have limited the depth of information collected. The survey was administered to managers of the participating centers rather than multiple healthcare specialists to achieve an institutional overview of the organization and delivery of nutritional care. Consequently, the findings mainly reflect the center-level perspective and may not fully capture potential variability in practices, perceptions and responsibilities among different healthcare professionals involved in nutritional care within the same institution. In addition, the self-administered nature of the survey did not allow an independent verification of the reported practices or their degree of implementation in routine clinical practice and may have introduced reporting bias and social desirability bias, as the reported information may reflect the intended or perceived organization of nutritional care rather than its actual implementation in routine clinical practice. Comparisons between public and private centers were exploratory and involved multiple statistical tests without formal adjustment for multiple comparisons. Therefore, the possibility of type I error cannot be excluded, and these findings should be interpreted cautiously.

This survey was conducted exclusively in the Lombardy Region, which may limit the generalizability of the results to other Italian regions and other international settings.

Healthcare organization, regional policies, availability of clinical nutrition services, referral pathways, and access to multidisciplinary resources may differ substantially across settings. Nevertheless, several gaps identified in this survey, including incomplete systematic screening, heterogeneous referral pathways, limited follow-up, and scarce use of PROs, QoL measures, and audit processes, may be relevant beyond the regional context. The findings may therefore help inform similar efforts aimed at strengthening nutritional care pathways in other healthcare settings. Although the survey was conducted exclusively in the Lombardy region, this regional focus was intentional as the survey was part of the preparatory phase for developing and implementing the regional PDTA for nutritional care in oncology. Lastly, the cross-sectional design and the absence of patient-level data did not allow us to assess temporal trends in nutritional care delivery or to examine whether the reported organizational practices were associated with actual nutritional, clinical or economic outcomes. Despite this limitation, we are planning a dedicated follow-up study to assess the effectiveness of the PDTA implementation in the Lombardy region over time.

## 5. Conclusions

This regional survey identified persistent gaps and unmet needs in the organization and delivery of nutritional care in oncology, particularly regarding systematic nutritional risk screening, timely nutritional assessment, follow-up, the use of patient-reported outcomes and quality of life measures, and quality assurance processes.

These findings informed the development of the regional Diagnostic–Therapeutic Care Pathway, which aims to promote a more homogeneous, structured, and simplified approach of nutritional care delivery across the Lombardy Region.

From a practical standpoint, the regional PDTA aims to optimize cancer patients’ nutritional care through systematic nutritional risk screening, assessment, and support in the context of a multidisciplinary approach. It also aims to integrate the use of technology, such as teleconsultation and telemedicine, to enhance delivery of care. Moreover, given the lack of assessment of PROs and QoL in patients with cancer, it also supports the evaluation of these aspects via validated tools as an integral part of the nutritional care process.

The implementation of the PDTA at the regional level may contribute to improving the consistency of nutritional care delivery for patients with cancer. Its impact on nutritional, clinical, quality of life and economic outcomes will need to be assessed prospectively. If the implementation proves effective, Lombardy Region experience might serve as a driver and possibly a guideline to identify areas for improvement also at the national level.

## Data Availability

The pooled data that support the findings of this research are available from the authors A.A., A.S., and R.C. upon reasonable request.

## Supplementary Materials

The following supporting information can be downloaded at: https://www.mdpi.com/article/10.3390/nu18152433/s1, Table S1: Full questionnaire and distribution of responses. Table S2: Comparison of nutritional care practices according to institutional (public vs. private) status.

## Abbreviations

The following abbreviations are used in this manuscript:

PDTA	Diagnostic–therapeutic care pathway
Q	Questions
PROs	patient-reported outcomes
QoL	quality of life
ATS	Local Health Agencies
ERAS	Enhanced Recovery After Surgery
MNTs	multidisciplinary nutritional teams
CNUs	Clinical Nutrition Units
