# Supplementary material for: Mapping Nutritional Assessment and Management in Oncology: Challenges and Perspectives from a Regional Survey
Source: Nutrients. 2026 Jul 25;18(15):2433. doi: 10.3390/nu18152433 (PMC13467806; doi:10.3390/nu18152433)
Supplement: Supplementary file 1 [file nutrients-18-02433-s001.zip › nutrients-4391238-supplementary.pdf]

Supplemental Table S1: Full questionnaire and distribution of responses.

Questions	*Answer N (%)*		
	Yes	*No*	*Not available*
**1. Does the Center provide nutritional care for cancer patients?**	**43 (78%)**	**12 (22%)**	**0 (0%)**
1.1 If yes, is nutritional care provided by the dietetic and clinical nutrition unit?	20 (47 %)	23 (53 %)	0 (0 %)
1.2 If yes, is nutritional care provided by the nutrition team (physician, dietitian, nurse, pharmacist)?	28 (65%)	15 (35 %)	0 (0 %)
1.3 If no, are there agreements with other active structures?	2 (17%)	10 (83%)	0 (0 %)
**2. Does the Centre have a procedure that describes the patient's nutritional pathway as an integral part of the oncological management?**	**31 (56%)**	**24 (44 %)**	**0 (0 %)**
2.1 If yes, is the procedure fully implemented?	24 (77 %)	7 (23 %)	0 (0 %)
2.2 If yes, does the procedure describe the criteria for patients' submission to the multidisciplinary discussion?	22 (71 %)	9 (29 %)	0 (0 %)
2.2.1 If yes, specify the submission criteria, n (%):			
- *Positive nutritional screening*	*10 (45 %)*		*0 (0 %)*
- *Weight loss/ low BMI/ reduced intakes*	*4 (18 %)*		
- *BMI < 20.5 or ≥ 30; Surgical Procedure; Patient with dysphagia or unable to feed himself/herself*	*3 (14 %)*		
- *Other*	*5 (23 %)*		
2.3 If yes, does the procedure describe the eligibility criteria for nutritional support (oral, parenteral, or enteral)?	22 (71 %)	9 (29 %)	0 (0 %)
**3. Is a nutritional prehabilitation program for surgery implemented at the Center?**	**27 (49%)**	**28 (51 %)**	**0 (0 %)**
**4. Is pre-operative immunonutrition administered at the Center?**	**19 (35%)**	**36 (65%)**	**0 (0 %)**
**5. At the Center, are all oncology patients currently subjected to nutritional screening?**	**23 (42%)**	**32 (58%)**	**0 (0 %)**
**6. Indicate through which methods the patient who tested positive to the screening is referred to the Clinical Dietetics and Nutrition Structure or Nutrition Team, n (%):**			
- *Mail and/or phone call*	*22 (40 %)*		*5 (9%)*
- *Computerized system*	*12 (22 %)*		
- *Computerized system and/or mail/phone call*	*7 (12 %)*		
- *Consulting*	*5 (9 %)*		
- *Outpatient appointment*	*2 (4 %)*		
- *Other*	*2 (4 %)*		

**7. Indicate the average time it takes for the Clinical Nutrition and Dietetics Department or Nutrition Team to take charge of a patient with serious nutritional risk, n (%)**			
- *24 hours*	*9 (16 %)*		*0 (0 %)*
- *48 hours*	*23 (42 %)*		
- *72 hours*	*11 (20 %)*		
- *>72 hours*	*12 (22 %)*		
**8. Nutritional screening is repeated regularly (at least every 1-2 weeks during hospitalization and at outpatient/DH follow-up visits) during the oncology pathway**	**17 (31%)**	**38 (69%)**	**0 (0 %)**
**9. The Center performs nutritional assessment on all cancer patients who test positive for screening**	**28 (51 %)**	**27 (49 %)**	**0 (0 %)**
9.1 If no, specify the criteria adopted to perform nutritional assessment, n (%):			
- *Clinical evaluation*	*12 (44 %)*		*4 (14 %)*
- *Weight loss and/or dysphagia*	*7 (26 %)*		
- *Multidisciplinary assessment*	*1 (4 %)*		
- *Guidelines*	*1 (4 %)*		
- *Inward patients*	*1 (4 %)*		
- *If NRS-2002 ≥5*	*1 (4 %)*		
**10. Select the nutritional assessment programming mode, n (%):**			
- *First + follow-up visit*	*29 (53 %)*		*0 (0 %)*
- *Only when the patient reports weight loss*	*11 (20 %)*		
- *First visit and repeat if weight loss*	*10 (18 %)*		
- *Only first visit*	*5 (9%)*		
**11. Does the Center have the anthropometric instruments necessary to carry out nutritional screening and evaluation?**	**44 (80%)**	**11 (20%)**	**0 (0 %)**
**12. Does the Center guarantee the provision of the first nutritional visit within one month of the diagnosis and the follow-up until the normalization of nutritional conditions?**	**35 (64%)**	**20 (36%)**	**0 (0 %)**
**13. Does the Center also provide follow-up via telemedicine/teleconsultation?**	**11 (20 %)**	**44 (80 %)**	**0 (0 %)**
13.1 If yes, is the use of tools or apps foreseen (e.g. for uploading food diary, recording weekly weights)?	1 (9 %)	10 (91 %)	0 (0 %)
**14. The center has a tool that enables the collection of patient-reported outcomes related to the nutritional management of cancer patients**	**8 (15 %)**	**47 (85 %)**	**0 (0 %)**
**15. The Center organizes training sessions (e.g., lectures, on-the-job training) for the personnel involved in oncological care**	**40 (73 %)**	**15 (27 %)**	**0 (0 %)**
**16. At the time of diagnosis of neoplasia, the Center provides the patient with informational materials (paper or digital) containing preliminary nutritional recommendations**	**29 (53 %)**	**26 (47 %)**	**0 (0 %)**
**17. From the initial stages of the treatment process, the Center implements nutritional educational interventions aimed at the cancer patient and the caregiver**	**35 (64 %)**	**20 (36 %)**	**0 (0 %)**

**18. The management of cancer patients with nutritional problems requires periodic evaluation of the quality of life using validated tools**	**11 (20 %)**	**44 (80 %)**	**0 (0 %)**
**19. At the Center, patient satisfaction is detected through a specific tool (e.g., customer satisfaction)**	**39 (71 %)**	**16 (29 %)**	**0 (0 %)**
**20. Periodic internal audits are conducted within the Center to verify the safety of nutritional care of the oncology patient using a specific checklist**	**8 (15 %)**	**47 (85 %)**	**0 (0 %)**
**21. The Center promotes awareness events related to appropriate eating habits and/or healthy lifestyles**	**38 (69 %)**	**17 (31 %)**	**0 (0 %)**

Data are presented as number (percentage). Percentages were calculated based on the row totals. For the main sections, percentages refer to the total number of centers that responded to the survey (n=55). For the subsections, percentages refer to the number of centers that provided a positive or negative response according to the corresponding main questions.

Supplemental Table S2: Comparison of nutritional care practices according to institutional (public vs private) status.

Questions	***Public (n=34) n (%)***			***Private (n=21) n (%)***			
	Yes	***No***	***NA***	***Yes***	***No***	***NA***	***p***
**1. Does the Center provide nutritional care for cancer patients?**	28 (82 %)	6 (18 %)	0 (0 %)	15 (71 %)	6 (29 %)	0 (0 %)	0.503
1.1 If yes, is nutritional care provided by the dietetic and clinical nutrition unit?	16 (57 %)	12 (43 %)	0 (0 %)	4 (27 %)	11 (73 %)	0 (0 %)	0.107
1.2 If yes, is nutritional care provided by the nutrition team (physician, dietitian, nurse, pharmacist)?	16 (57 %)	12 (43 %)	0 (0 %)	12 (80 %)	3 (20 %)	0 (0 %)	0.186
1.3 If no, are there agreements with other active structures?	1 (17 %)	5 (83 %)	0 (0 %)	1 (17 %)	5 (83 %)	0 (0 %)	1.000
**2. Does the Centre have a procedure that describes the patient's nutritional pathway as an integral part of the oncological management?**	20 (59 %)	14 (41 %)	0 (0 %)	11 (52 %)	10 (48 %)	0 (0 %)	0.781
2.4 If yes, is the procedure fully implemented?	16 (80 %)	4 (20 %)	0 (0 %)	8 (73 %)	3 (27 %)	0 (0 %)	0.676
2.5 If yes, does the procedure describe the criteria for patients' submission to the multidisciplinary discussion?	14 (70 %)	6 (30 %)	0 (0 %)	8 (73 %)	3 (27 %)	0 (0 %)	1.000
2.2.1 If yes, specify the submission criteria, n (%):							
- *Positive nutritional screening*	7 (50 %)		0 (0 %)	3 (21 %)		0 (0 %)	0.082
- *Weight loss/ low BMI/ reduced intakes*	3 (21 %)			1 (7 %)			

- *BMI < 20.5 or ≥ 30; Surgical Procedure; Patient with dysphagia or unable to feed himself/herself*	0 (0 %)			3 (21 %)			
- *Other*	4 (29 %)			1 (7 %)			
2.6 If yes, does the procedure describe the eligibility criteria for nutritional support (oral, parenteral, or enteral)?	15 (75 %)	5 (25 %)	0 (0 %)	7 (64 %)	4 (36 %)	0 (0 %)	0.683
**3. Is a nutritional prehabilitation program for surgery implemented at the Center?**	21 (62 %)	13 (38 %)	0 (0 %)	6 (29 %)	15 (71 %)	0 (0 %)	0.026
**4. Is pre-operative immunonutrition administered at the Center?**	18 (53 %)	16 (47 %)	0 (0 %)	1 (5 %)	20 (95 %)	0 (0 %)	<0.001
**5. At the Center, are all oncology patients currently subjected to nutritional screening?**	16 (47 %)	18 (53 %)	0 (0 %)	7 (33 %)	14 (67 %)	0 (0 %)	0.403
**6. Indicate through which methods the patient who tested positive to the screening is referred to the Clinical Dietetics and Nutrition Structure or Nutrition Team, n (%):**							
- *Mail and/or phone call*	12 (35 %)		3 (9 %)	10 (48 %)		2 (9%)	<0.001
- *Computerized system*	10 (29 %)			2 (10 %)			
- *Computerized system and/or mail/phone call*	4 (12 %)			3 (14 %)			
- *Consulting*	2 (6 %)			3 (14 %)			
- *Outpatient appointment*	2 (6 %)			0 (0 %)			
- *Other*	1 (3 %)			1 (5 %)			
**7. Indicate the average time it takes for the Clinical Nutrition and Dietetics Department or Nutrition Team to take charge of a patient with serious nutritional risk, n (%)**							
- *24 hours*	6 (18 %)		0 (0 %)	3 (14 %)		0 (0 %)	<0.001
- *48 hours*	15 (44 %)			8 (38 %)			
- *72 hours*	6 (18 %)			5 (24 %)			
- *>72 hours*	7 (21 %)			5 (24 %)			
**8. Nutritional screening is repeated regularly (at least every 1-2 weeks during hospitalization and at outpatient/DH follow-up visits) during the oncology pathway**	14 (41 %)	20 (59 %)	0 (0 %)	3 (14 %)	18 (86 %)	0 (0 %)	0.042
**9. The Center performs nutritional assessment on all cancer patients who test positive for screening**	21 (62 %)	13 (38 %)	0 (0 %)	7 (33 %)	14 (67 %)	0 (0 %)	0.054
9.2 If no, specify the criteria adopted to perform nutritional assessment, n (%):							
- *Clinical evaluation*	6 (46 %)		2 (15 %)	6 (43 %)		2 (15 %)	0.061

- *Weight loss and/or dysphagia*	4 (31 %)			3 (21 %)			
- *Multidisciplinary assessment*	1 (8 %)			0 (0 %)			
- *Guidelines*	0 (0 %)			1 (7 %)			
- *Inward patients*	0 (0 %)			1 (7 %)			
- *If NRS-2002 ≥5*	0 (0 %)			1 (7 %)			
**10. Select the nutritional assessment programming mode, n (%):**							
- *First + follow-up visit*	21 (62 %)		0 (0 %)	8 (38 %)		0 (0 %)	<0.001
- *Only when the patient reports weight loss*	5 (15 %)			6 (29 %)			
- *First visit and repeat if weight loss*	5 (15 %)			5 (24 %)			
- *Only first visit*	3 (9 %)			2 (10 %)			
**11. Does the Center have the anthropometric instruments necessary to carry out nutritional screening and evaluation?**	27 (79 %)	7 (21 %)	0 (0 %)	17 (81 %)	4 (19 %)	0 (0 %)	1.000
**12. Does the Center guarantee the provision of the first nutritional visit within one month of the diagnosis and the follow-up until the normalization of nutritional conditions?**	24 (71 %)	10 (29 %)	0 (0 %)	11 (52 %)	10 (48 %)	0 (0 %)	0.249
**13. Does the Center also provide follow-up via telemedicine/teleconsultation?**	7 (21 %)	27 (79 %)	0 (0 %)	4 (19 %)	17 (81 %)	0 (0 %)	1.000
13.2 If yes, is the use of tools or apps foreseen (e.g. for uploading food diary, recording weekly weights)?	1 (14 %)	6 (86 %)	0 (0 %)	0 (0 %)	4 (100 %)	0 (0 %)	1.000
**14. The center has a tool that enables the collection of patient-reported outcomes related to the nutritional management of cancer patients**	7 (21 %)	27 (79 %)	0 (0 %)	1 (5 %)	20 (95 %)	0 (0 %)	0.136
**15. The Center organizes training sessions (e.g., lectures, on-the-job training) for the personnel involved in oncological care**	27 (79 %)	7 (21 %)	0 (0 %)	13 (62 %)	8 (38 %)	0 (0 %)	0.215
**16. At the time of diagnosis of neoplasia, the Center provides the patient with informational materials (paper or digital) containing preliminary nutritional recommendations**	17 (50 %)	17 (50 %)	0 (0 %)	12 (57 %)	9 (43 %)	0 (0 %)	0.782
**17. From the initial stages of the treatment process, the Center implements nutritional educational interventions aimed at the cancer patient and the caregiver**	23 (68 %)	11 (32 %)	0 (0 %)	12 (57 %)	9 (43 %)	0 (0 %)	0.565
**18. The management of cancer patients with nutritional problems requires periodic evaluation of the quality of life using validated tools**	8 (24 %)	26 (76 %)	0 (0 %)	3 (14 %)	18 (86 %)	0 (0 %)	0.319
**19. At the Center, patient satisfaction is detected through a specific tool (e.g., customer satisfaction)**	23 (68 %)	11 (32 %)	0 (0 %)	16 (76 %)	5 (24 %)	0 (0 %)	0.556

**20. Periodic internal audits are conducted within the Center to verify the safety of nutritional care of the oncology patient using a specific checklist**	5 (15 %)	29 (85 %)	0 (0 %)	3 (14 %)	18 (86 %)	0 (0 %)	1.000
**21. The Center promotes awareness events related to appropriate eating habits and/or healthy lifestyles**	25 (74 %)	9 (26 %)	0 (0 %)	13 (62 %)	8 (38 %)	0 (0 %)	0.386

Data are presented as number (percentage). Percentages were calculated based on the row totals. For the main sections, percentages refer to the total number of centers that responded to the survey (Private: n=21; Public: n=34). For the subsections, percentages refer to the number of centers that provided a positive or negative response according to the corresponding main questions.
